# Low Tibial and Fibular Osteotomy for Treating Varus-Type Post-Traumatic Ankle Osteoarthritis: A Case Report

**DOI:** 10.5704/MOJ.2007.025

**Published:** 2020-07

**Authors:** MZ Chilmi, AT Desnantyo, L Widhiyanto, BC Wirashada

**Affiliations:** Department of Orthopaedic and Traumatology, Universitas Airlangga, Surabaya, Indonesia

**Keywords:** low tibial osteotomy, post-traumatic ankle osteoarthritis, tibial-ankle surface angle (TAS), ankle arthritis

## Abstract

In Indonesia, arthrodesis becomes a choice of treatment in the absence of ankle arthroplasty implants for young adults. Arthrodesis on ankle osteoarthritis (OA) often leads to functional impairment. Low tibiofibular osteotomy is an alternative and it has been known to be the preferable option for those in the productive-ages. A 22-year-old male with a previous history of a motorbike accident, operated eight years ago, came with persistent pain on the left ankle that has worsened over the years. Plain radiography with a tibial-ankle surface angle (TAS) of 74^o^ (normally 88^o^-93^o^) indicated varus deformity. Osteotomy was performed on distal tibia above the syndesmotic joint, as well as on the middle third of fibula. Open wedge osteotomy of the tibia was corrected until the normal TAS angle was reached by fluoroscopy. Cortical allograft was used to fill the osteotomy gap. Instrumentation was performed using a clover leaf® plate with 6 screws insertion for fixation stability. All results were satisfactory. Twelve weeks post-operatively, the patient was performing activities normally. Four-month post-operative radiological evaluation showed fusion of graft and the angle of TAS of 89°. Post-operative functional assessment using the American Academy of Orthopaedic Surgeon (AAOS) Foot and Ankle Measurement (FAM) questionnaires showed significant improvement (pre 89, post 38).

## Introduction

Ankle osteoarthritis (OA) occurs in one percent of the world adult population. In contrast to OA in the pelvis and knee, ankle OA is often caused by trauma^1^. Malunion and intra-articular fractures are the common causes. Flatfoot in the adult is a predisposing factor of idiopathic valgus osteoarthritis and varus osteoarthritis, or supramalleolar abnormality^2^.

Treatments include conservative bracing, pain relief injection, while surgeries include tibial and fibular osteotomy, arthroscopy and ankle arthroplasty or ankle arthrodesis^2^. Arthroplasty has a good outcome but not suitable for patients under 50 years old. Low osteotomy is a procedure to preserve the joints previously in asymmetric valgus deformity or ankle arthritis that forms varus deformity. This procedure aims to reduce pain, harmful mechanics of joints and inhibits joint degeneration^1^.

Angular deformity was evaluated using Tibial Ankle Surface (TAS) angle or tibial-distal-lateral angle, talar-tilted angle, and Talar-Lateral Surface (TLS) angle. TAS connects the tibial axis with tibial ceilings (normally 89^o^); the talus slope is the angle between tibial-ceiling and the talar dome (normal <4^o^); TLS connects the tibial axis and lines of anterior aspect and posterior aspect of tibial ceiling (normally 81.5^o^)^2^.

Studies showed low tibial osteotomy decreases pain, improves function, and corrects varus deformity^3^. Cheng reported satisfactory results in 18 low tibial osteotomy patients^4^. Knupp *et al* supported with 94 ankle OA patients who underwent low tibial osteotomy with significant statistical and clinical pain relief calculated by American Orthopaedic Foot and Ankle Society (AOFAS) Ankle-Hindfoot score after follow-up for 3.6 years^1^. This report emphasised the advantage of the low tibiofibular osteotomy.

## Case Report

We report a 22-year-old male with left ankle pain since the last two years before admission. Pain is especially felt in the morning, while moving from sit to stand position; sometimes the patient is also unable to walk. He had a motorcycle accident eight years ago, was diagnosed with ankle fracture AO 44B2.1 and underwent internal fixation. Implant removal was done a year later.

**Fig. 1:**
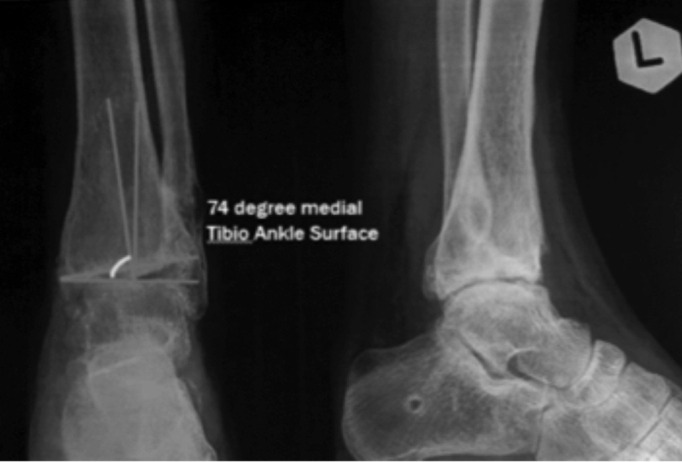
Pre-operative condition. Left ankle pre-operative imaging, showed varus deformity with TAS angle 74^o^ (internal documentation).

Upon admission to our hospital, we performed thorough anamnesis, physical examination of ankle range of motion (ROM), and radiologic imaging. Left ankle varus deformity, positive talar tilt test, tenderness, and limited ROM was observed during physical examination. Radiologically, stage 3 ankle OA was seen. Varus deformity (TAS) was confirmed to be 74° (normal 88^o^-93^o^) (Fig. 1). No history of diabetes mellitus, corticosteroid, and alcohol consumption were obtained. The pre-operative questionnaire of AAOS FAM was taken to measure ankle-foot function objectively.

Few procedures have been described and although it has the disadvantage of joint motion loss, ankle arthrodesis is still preferred by experts. In order to maintain the joint motion, the authors performed low-tibial osteotomy.

**Fig. 2:**
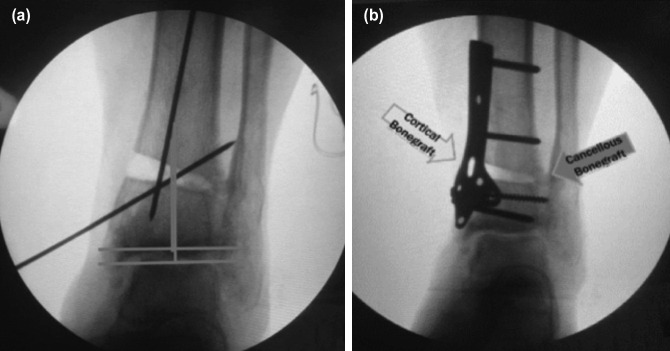
Intra-operative imaging. (a) Open wedge osteotomy on distal tibia until normal TAS angle reached; K-wire used for temporary fixation. (b) Cortical allograft used to fill the gap and cancellous bone graft, fixated using plate and screw (internal documentation).

The open wedge osteotomy was performed on the left distal tibia above the syndesmotic joint as well as on the middle third of the fibula under fluoroscopic guidance until normal TAS was achieved (Fig. 2a).

After the normal TAS was achieved, cortical allograft and cancellous bovine bone graft were used to fill the osteotomy gap on distal tibia, the graft were temporarily stabilised with wire. For the final fixation, a clover-leaf^®^ plate with six screws were used (Fig. 2b).

**Fig. 3:**
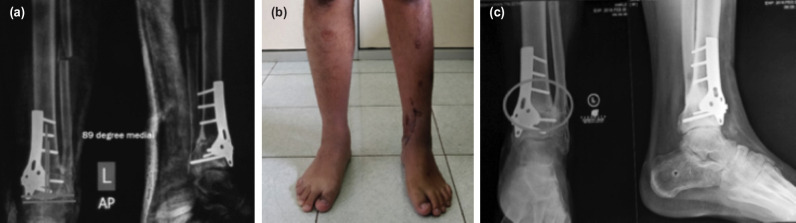
Post-operative follow-up (a) Post-operative imaging of left ankle with TAS angle of 89^o^. (b) Four weeks post-operative clinical condition, varus deformity is corrected. (c) Four months post-operative evaluation showed graft fusion and normal TAS angle (internal documentation).

Immediate post-operative evaluation showed satisfactory 89o TAS and reduced AAOS FAM disability index scores. The scale improved from 89 to 38 post-operatively which indicated significant improvement of patient’s ankle-foot function (Fig. 3a). Four weeks post-operative clinical condition showed healed scar and corrected varus deformity (Fig. 3b).

Full weight-bearing and return to activities were achieved at 12 weeks post-operative, while radiological union with complete graft fusion was achieved 4 months post-operative (Fig. 3c).

## Discussion

Post-traumatic ankle osteoarthritis is very rare. Varus type osteoarthritis of the ankle originates from both traumatic and idiopathic causes. Varus type osteoarthritis can be classified into stage I (no joint space narrowing yet sclerosis and osteophyte presents), stage II (reduction of medial joint space), stage III (joint space obliterated medial aspect and contact between adjacent subchondral bone), or stage IV (loss of joint space with connection between tibia and talus)^4^. Treatment of ankle OA in the severe stage is debatable. Ankle arthrodesis often becomes the expert’s choice due to the unavailability of arthroplasty implant for the ankle in many countries.

Hongmou *et al* reported the treatment of OA at mid-ankle to maintain half or more tibiotalar joint surface is challenging and controversial. Supramalleolar osteotomy (SMOT) is an effective procedure for the treatment of an asymmetric ankle OA, aimed at realigning the mechanical axis of the ankle joint^1, 4, 5^.

The minimally invasive posteromedial approach in combination with minimally invasive plate osteosynthesis (MIPO) may minimise soft tissue dissection to reduce wound complications. In such cases, the osteotomy is performed through fibula, tibia, and synostosis in between. The anteromedial incision to complement the medial part of the osteotomy. The elastic plate can be used to protect the neurovascular bundle (protecting anterior tibial artery and peroneal nerve)^5^.

Takakura *et al* reported 18 patients with OA ankles who underwent low tibial osteotomy surgery, which showed satisfactory results at 6 years follow up. In another study, there was an improvement in radiological features where the TAS angle was corrected from 82.7^o^ to 98.2^o^ and TLS angle from 78.5^o^ to 84.7^o^. Varus angle also improved from 7.3^o^ to 5.0^o^ post-operatively^4^.

Tibial open wedge osteotomy is recommended rather than lateral closing wedge osteotomy in treating the varus deformity. The complexity of the anterolateral compartment of the knee becomes the main risk factor for lateral muscle weakness in many reported lateral closing wedge procedure.

Due to its advantage of preserved joint motion compared to arthrodesis, low tibial osteotomy could be considered as a surgical choice for severe ankle osteoarthritis in productive age patients. Fibular osteotomy provides an additional advantage in ankle joint angle correction^1-3, 5^. The result is good and we achieved the main surgical goal of this technique.
